# Synovial Sarcoma of the Head and Neck: A Single Institution Review

**DOI:** 10.1155/2017/2016752

**Published:** 2017-06-05

**Authors:** Vancheswaran Gopalakrishnan, Behrang Amini, Michael J. Wagner, Erica N. Nowell, Alexander J. Lazar, Patrick P. Lin, Robert S. Benjamin, Dejka M. Araujo

**Affiliations:** ^1^Division of Surgical Oncology, The University of Texas MD Anderson Cancer Center, 1515 Holcombe Blvd., Houston, TX 77030, USA; ^2^Division of Epidemiology, Human Genetics and Environmental Sciences, The University of Texas School of Public Health, 1200 Pressler St., Houston, TX 77030, USA; ^3^Division of Diagnostic Radiology-Musculoskeletal Imaging, The University of Texas MD Anderson Cancer Center, 1515 Holcombe Blvd., Houston, TX 77030, USA; ^4^Division of Cancer Medicine, The University of Texas MD Anderson Cancer Center, 1515 Holcombe Blvd., Houston, TX 77030, USA; ^5^Department of Sarcoma Medical Oncology, The University of Texas MD Anderson Cancer Center, 1515 Holcombe Blvd., Houston, TX 77030, USA; ^6^Department of Pathology, The University of Texas MD Anderson Cancer Center, 1515 Holcombe Blvd., Houston, TX 77030, USA; ^7^Department of Orthopedic Oncology, The University of Texas MD Anderson Cancer Center, 1515 Holcombe Blvd., Houston, TX 77030, USA

## Abstract

**Background:**

The prognosis and clinical characteristics of head and neck synovial sarcomas (HNSS) are unclear. Herein, we present an update using a cohort of patients treated at our institution.

**Methods:**

We performed a retrospective chart review of 44 patients diagnosed with primary HNSS between March 1990 and June 2012. Overall survival (OS) and progression-free survival (PFS) curves were estimated and hazard ratios (HRs) were calculated.

**Results:**

The entire cohort's median PFS was 4.6 years, and 20 of the 44 (45%) patients developed either local or distant recurrence. Tumor size ≥ 5 cm (*p* = 0.008, HR = 4.69; 95% CI = 1.34–16.38) and a primary presentation in the soft tissues of the neck (*p* = 0.04, HR = 2.41; 95% CI = 1.003–5.82) were associated with significantly worse PFS. The OS and PFS of patients who received definitive local therapy versus those who received additional adjuvant systemic therapy did not differ significantly.

**Conclusion:**

Despite the treatment challenges associated with HNSS, our cohort of patients had a better prognosis than one might expect in this unfavorable anatomical location. Our findings suggest that tumor size and site are predictive of PFS and that wide surgical excision is of vital importance, since traditional cytotoxic chemotherapy has limited efficacy at this site.

## 1. Introduction

Synovial sarcomas (SS) are distinct soft tissue sarcomas that have a predilection for young and middle-aged adults [1, 2]. “Synovial” indicates these tumors' close microscopic resemblance to the synovium, but SS in fact originate from pluripotent mesenchymal cells and not synovial structures [3, 4]. SS account for approximately 8% of all soft tissue sarcomas and most often affect the extremities [3]. Despite the existence of histologic grading criteria based on mitotic index and tumor necrosis, most SS are considered high-grade tumors [5, 6]. They can be classified as monophasic or biphasic based on the presence of either a spindle cell component or both an epithelial and spindle cell component, respectively; the monophasic type is more common. Approximately 90% of SS have the chromosomal translocation t(X; 18), which results in the formation of a fusion product between the synaptotagmin 1 gene,* SYT1*, on chromosome 18 and the SSX family member 1 gene,* SSX1*, or SSX family member 2 gene,* SSX2*, on chromosome X [2, 6, 7]. SS frequently metastasize to the lungs and are associated with 10-year survival rates of <50% [8–10]. Wide local excision followed by adjuvant radiation and/or chemotherapy is the recommended treatment modality [1].

SS rarely occur in the head and neck and comprise only 2.5–3.5% of all sarcomas in this region [2, 11]. Compared with other SS, head and neck SS (HNSS) are believed to have a greater potential for both regional and distant metastasis, with the primary mode of spread being hematogenous [12, 13]. HNSS were first reported by Jernstorm in 1954 [14]; since then, several small studies have outlined the prognostic factors for this unique sarcoma. HNSS are more common in men and more often present during middle age [15]. There is a lack of consensus regarding the most common sites of primary HNSS; both the upper aerodigestive tract and the soft tissues of the neck have been reported separately [2, 7, 15–17]. The most common clinical presentation of HNSS is a painless mass that is occasionally associated with hoarseness, dysphagia, odynophagia, and/or bleeding [17–21]. Like other SS, HNSS are often treated with wide local excision plus adjuvant radiation and/or chemotherapy [15].

There is a lack of clear evidence on the prognosis of HNSS. Clinicians in the field frequently assume that HNSS have a worse prognosis than primary SS at other sites do. However, several reports indicate that the prognosis of HNSS is better than that of SS arising in the extremities (overall survival: 47%–82%) [16]. Therefore, given the paucity of published information and the existence of conflicting reports regarding the prognosis of HNSS, we sought to present an update on the clinical characteristics and survival of patients with this malignancy.

## 2. Patients and Methods

This retrospective cohort study was approved by the Institutional Review Board. From a sampling of 648 patients who presented to our institution with a diagnosis of SS from March 1990 through June 2012, we identified 44 patients (6.7%) who had a primary HNSS in the head and neck region and were treatment-naïve. Patients who had a primary tumor at a different site with metastasis to the head or neck were not included in the analysis. These 44 patients' clinical and demographic information, including age at diagnosis, gender, race, tumor size, tumor subtype (monophasic or biphasic), surgical margins (if applicable), presence of the* SS18-SSX* fusion gene, and therapeutic regimen for the primary tumor, was extracted from the institutional medical records and database. Pretreatment tumor sizes were calculated at baseline by an experienced musculoskeletal radiologist. Therapies were classified as “definitive local therapy” if the patient had definitive therapy (surgery and/or radiotherapy) only or “definitive local therapy + systemic therapy” if the patient received adjuvant systemic therapy (most patients received the combination of doxorubicin and ifosfamide) in addition to definitive local therapy.

Progression-free survival (PFS) and overall survival (OS) estimates were calculated using the Kaplan-Meier method. PFS duration was defined as the interval between the date of the diagnosis of the primary tumor and the date of the appearance of either a local or a distant recurrence. OS duration was defined as the interval between the date of diagnosis of the primary tumor and the date of death. Patients who were lost to follow-up during the study period were censored at their date of last contact. The effect of various factors on survival was determined using the log-rank test. Hazard ratios were estimated using the Cox proportional-hazards model. All analyses were done using STATA 14 (StataCorp, College Station, TX) and GraphPad Prism v6 for Windows (GraphPad Software, La Jolla, CA). Statistical significance testing was two-sided at a type 1 error rate (*α*) of 0.05.

## 3. Results

The cohorts' median age at diagnosis was 31.5 years, and most patients were men (77%) and white (75%). Pretreatment measurements of the primary tumors were available for 38 of the 44 patients. We found an even distribution of tumor sizes <5 cm and ≥5 cm, with approximately 53% of patients presenting with a primary tumor ≥5 cm. Primary tumors were found at several sites but were predominant in the soft tissues of the neck (34%). Of the 33 patients for whom the pathological subtype was available, 19 (58%) had the monophasic subtype, 12 (36%) had the biphasic subtype, and 2 (6%) had the poorly differentiated subtype. The calcified subtype was not identified in this cohort. Treatment information (1st line of therapy) was available for 41 patients; of these, 25 (61%) received systemic therapy in addition to definitive local therapy, 15 (37%) received local therapy only, and 1 (2%) received systemic therapy only (Table 1).

**Figure 1:**
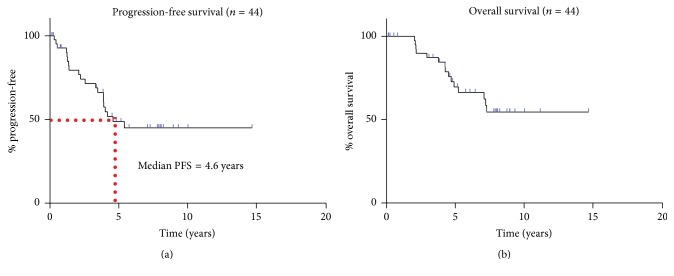
Kaplan-Meier survival curves showing PFS (a) and OS (b) for the entire cohort (*n* = 44) of head and neck synovial sarcoma patients. The median PFS was 4.6 years. Median OS not reached.

20 of the 44 patients (45%) developed either local or distant recurrence. The median PFS was 4.6 years, whereas the median OS had not been reached (Figure 1). PFS rates were 79.4% (95% confidence interval [CI] = 63%–89.2%), 48.7% (95% CI = 31.8%–63.6%), and 44.9% (95% CI = 28.1%–60.4%) at 2, 5, and 10 years, respectively. Even more remarkable, OS rates were 100%, 69.5% (95% CI = 48%–79.4%), and 54.5% (95% CI = 35.5%–70.1%) at 2, 5, and 10 years, respectively.

**Figure 2:**
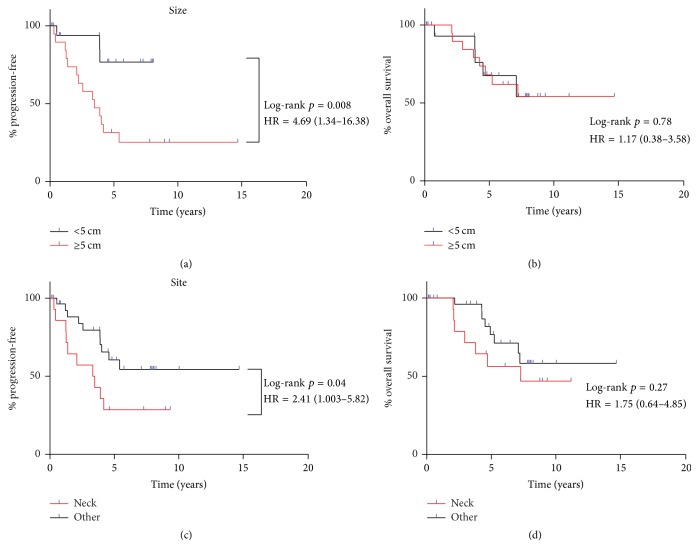
Kaplan-Meier curves revealed a significant difference in PFS between patients grouped according to the size of the primary tumor (a) and site of the primary tumor (c). There was no significant difference in OS between patients grouped according to the size of the primary tumor (b) and site of the primary tumor (d).

The median PFS of patients with tumors ≥5 cm (3.33 years) was significantly lesser than that of patients with tumors <5 cm (median PFS not reached; log-rank *p* = 0.008). Additionally, the PFS of patients with primary tumors in the soft tissues of the neck (3.47 years) was also significantly less than that of patients with primary tumors at other sites (median PFS not reached; log-rank *p* = 0.04). No differences were noted between men (5.41 years) and women (3.47 years; log-rank *p* = 0.21) and between patients who received definitive local therapy only (5.41 years) versus those who also received systemic therapy (4.58 years; log-rank *p* = 0.78). We also found no differences in terms of OS (Figure 2 and Table 2).

Treatment modalities were evenly distributed by tumor size. Among the 20 patients with primary tumors ≥5 cm, eight received definitive local therapy only, whereas 11 also received additional systemic therapy; one patient received systemic therapy only. On the other hand, among the 18 patients with primary tumors <5 cm, 8 received definitive local therapy only, whereas 10 also received systemic therapy (*p* = 1). We found no significant differences in PFS or OS between these subgroups (data not shown).

The univariate Cox proportional-hazards model revealed that tumor size ≥5 cm and a primary tumor in the neck were associated with an increased hazard for progression (hazard ratio [HR] = 4.69, 95% CI = 1.34–16.38 and HR = 2.41, 95% CI = 1.00–5.82, resp.) and death (HR = 1.17, 95% CI = 0.38–3.58 and HR = 1.75, 95% CI = 0.64–4.85, resp.). Patients who received systemic therapy in addition to definitive local therapy had an increased but nonsignificant hazard for both progression (HR = 1.14, 95% CI = 0.45–2.91) and death (HR = 1.16, 95% CI = 0.39–3.46) (Table 3).

## 4. Discussion

SS are rare in the head and neck region [4]. In the present study, we could identify only 44 patients over 22 years who presented to our institution with primary HNSS. Given the treatment challenges associated with the head and neck region, one might reasonably expect the prognosis of HNSS to be poor. However, our findings indicate that HNSS in fact have a relatively good prognosis compared with other SS.

Importantly, patients with HNSS had good survival rates. Similar OS rates have been reported for 167 HNSS patients in a recent Surveillance, Epidemiology, and End Results Program database analysis [16]. A prior study from our institution, which included 42 patients with not only primary tumors but also recurrent and metastatic tumors, reported a similar 5-year OS rate (72%) [2]. Several other studies have also reported 5-year OS rates ranging from 40% to 70% [7, 15, 17, 19] (Table 4).

In the present study, primary tumor size was a predictor of progression, as evidenced by significantly different Kaplan-Meier PFS curves (*p* = 0.008) and a significant HR (HR = 4.69, 95% CI = 1.34–16.38). The mean and median tumor sizes for the 38 patients on whom tumor size data was available were 5.34 cm and 4.85 cm, respectively, which justified the choice of 5 cm as the cutoff value for identifying tumors as either large or small. There has been considerable debate regarding the choice of the tumor size cutoff value for HNSS. Although most studies have utilized the 5 cm cutoff [2, 15, 16], a few others have found merit in using the 4 cm cutoff [7, 20]. Regardless of the value used, larger tumors have been found to be associated with poorer OS and PFS, which is consistent with our results.

Given the general predilection HNSS has for young men, our study cohort's median age at diagnosis (31.5 years) and male predominance are not surprising. In the present study, we found no significant differences in either PFS or OS between patients grouped by sex or by age (<31.5 years versus ≥31.5 years). There is little evidence that race influences the survival of HNSS patients. In the present study, most patients were white, and race did not influence either PFS or OS.

HNSS is known to affect several sites within the head and neck region. The parapharyngeal space in the neck is the most common site of HNSS presentation, and tumors in this area generally arise from the paraspinal muscles [11]. Within this space, the upper aerodigestive tract is not only the most common site of these tumors' presentation but also the site whose tumors have the best prognosis [2, 20]. The patients in our cohort also presented with primary tumors at several sites in the head and neck. The median PFS was least for patients with tumors arising in the soft tissues of the neck (3.33 years) followed by tumors in the face (3.38 years) and larynx (3.88 years). However, this might be an effect of sample size, as the number of patients in each site subgroup was quite small. Nevertheless, the PFS of patients who presented with a primary tumor in the soft tissues of the neck was significantly lesser than that of all other patients.

Traditionally, histologic subtype has not been known to influence survival in HNSS patients. Our results are concordant, with no major differences seen with PFS or OS based on subtype. Harb et al. reported that patients with monophasic tumors had significantly longer time to progression than patients with biphasic tumors did, but they also noted that this finding may have been confounded by tumor size [2]. In our study, tumors <5 cm and ≥5 cm were evenly distributed between patients with monophasic tumors and those with biphasic tumors, thereby accounting for any potential confounding by tumor size.

The* SS18-SSX* fusion gene aids in the histopathological diagnosis of SS, especially since it is often the disease's sole cytogenetic abnormality. Some studies have reported that patients with this fusion gene have a favorable prognosis [22, 23]. In our study, information about the* SS18-SSX* fusion gene was available for 19 of 44 patients. The remaining patients were not tested for this gene; therefore, we were unable to draw any definitive conclusions about its effect on prognosis. Among the patients who tested positive, only 6 had information available on whether the fusion gene was driven by the SSX1 or SSX2 gene. Of note, the median PFS of patients with the fusion gene was 3.9 years, which was less than that of the entire cohort. In addition, PFS significantly differed by size in this subgroup; the median OS was not reached.

Historically, grade does not trigger differences in treatment at our institution and since definitive grading on the resection is precluded by neoadjuvant therapy, and needle biopsy grading is fraught with potential undergrading, FNCLCC grades are not usually applied. The role of fusion variants (SS18-SSX1 versus SS18-SSX2) and grade in association with clinical outcomes is complex in synovial sarcoma. Kawai et al. proposed in 1998 with a cohort of 45 synovial sarcoma patients that SSX1 was associated with the biphasic phenotype and that SSX2 was associated with better outcomes [22]. With a much larger cohort of 243 in 2002, Ladanyi et al. were able to replicate these same findings [23]. In 2004, the French sarcoma group that established and validated the FNCLCC grading system showed in a cohort of 165 synovial sarcoma patients that SSX1 and SSX2 had equivalent outcomes when FNCLCC grade was considered in a multivariate analysis [24]. Ladanyi et al. independently accepted these results and recommended further studies [25, 26]. In 2008, Yoshikawa and colleagues examined 108 patients with fusion data and found no effect of SSX variant, with tumor size having the most consequential hazard ratio on multivariate analysis with grade showing very modest effects. In 2013, Ren et al. demonstrated that SSX2 variants, FNCLCC grade 2, and low UICC stage all had independent prognostic implications in a multivariate analysis of 88 patients [27]. Finally, Stegmaier et al., in a cohort of 243 pediatric patients (84 with localized disease and SSX type known), reported that neither grade nor fusion type was independently associated with outcome, but rather male sex and tumor size were implicated in multivariate analysis [28].

The most common treatment for HNSS is surgery, followed by radiotherapy. In their study of 167 HNSS patients, Mallen-St. Clair et al. reported that 150 patients (89.8%) had surgery and 108 (64.7%) had radiotherapy [16]. In general, negative margins following sarcoma resection are associated with a favorable prognosis [29, 30]. In SS patients, negative margins have been reported to be strongly associated with better PFS [24]. Surgical margin status was available for only 20 patients in our study, and we found no significant differences in either progression status or PFS between patients with positive or negative margins. It is possible that radiation may have mitigated the effects of positive margins in these patients.

In general, chemotherapy for HNSS should be given only in the presence of poor prognostic factors such as large tumor size (>5 cm) or an unfavorable site of presentation [2]. Despite being counterintuitive, patients who received both local and systemic therapy had poorer PFS than patients who received local therapy only did. The numbers of patients grouped by tumor size or site were evenly distributed among the treatment categories. It is quite possible in this retrospective analysis that there was a hidden selection bias toward administration of chemotherapy to patients who presented with more unfavorable characteristics, such as abutment against a vital anatomic structure, which may have led to thinner margins.

Timing of chemotherapy and/or radiotherapy did not seem to affect either PFS or OS in our cohort (Supplemental Table S1 in the Supplementary Material available online at https://doi.org/10.1155/2017/2016752). All but one of the patients who recurred underwent further therapy (either local or systemic therapy or both). There were no differences in survival based on choice of subsequent therapeutic regimen after recurrence (Supplemental Table S2).

Our study had several potential limitations. Because the study was retrospective, we could not control the collection of data, and missing data in some instances meant that the study lacked power to detect meaningful differences. Although we assembled a unique cohort, the overall sample size was still quite small, but it still may be relatively large for the study of a rare tumor. The distribution of treatments could not be assumed to be random, and it is conceivable that patients with more unfavorable tumors were treated more aggressively. In addition, we could not perform a multivariate analysis owing to the lack of an adequate number of events (progression, death). Lastly, because MD Anderson is a tertiary referral center, the study is susceptible to selection bias, and its results might not be truly representative of the overall HNSS patient population.

## 5. Conclusion

HNSS are a unique oncologic entity that should be treated as such. To our knowledge, our study presents the largest cohort of treatment-naïve HNSS patients to date. The relatively high 5- and 10-year OS and PFS rates of patients with HNSS suggest that the prognosis of these tumors is fair to good despite the significant treatment challenges associated with the head and neck region. We also appreciate that tumor size is the single most important predictor of progression. Importantly, no differences in PFS and OS rates by treatment were seen in patients with small (<5 cm) or large (≥5 cm) primary tumors. Therefore, given the overall poor prognosis associated with SS owing to its unique biological behavior, we recommend that all HNSS patients be treated in a sarcoma center and that treatment (surgery with or without radiotherapy and/or chemotherapy) be decided on a case-by-case basis. Further studies are warranted to validate our results and to identify the molecular characteristics driving not only oncogenesis but also therapy response in HNSS.

## Supplementary Material

Table S1: Comparison of median PFS and OS times by timing of radiation and chemotherapy in patients with head and neck synovial sarcomas.
Table S2: Comparison of median overall survival time by therapeutic regimen in patients with head and neck synovial sarcoma who were diagnosed with recurrent disease.

## Figures and Tables

**Table 1:** Patient characteristics.

Variable	*N* (%)
*Total cohort *	44 (100)
*Median age at diagnosis, years*	31.5
*Sex*	
Male	34 (77)
Female	10 (23)
Total	44
*Race*	
White	33 (75)
Other	11 (25)
Total	44
*Tumor size*	
≥5 cm	20 (53)
<5 cm	18 (47)
Total	38
*Subtype*	
Monophasic	19 (58)
Biphasic	12 (36)
Poorly differentiated	2 (6)
Total	33
*Surgical margin*	
Positive	6 (30)
Negative	14 (70)
Total	20
*Fusion gene (SS18-SSX)*	
Present	19 (43.2)
Not tested	26 (59.1)
Total	44
*Site*	
Face	8 (18)
Pharynx/larynx	12 (27)
Neck	15 (34)
Oral cavity	5 (11)
Other	4 (9)
Total	44
*Treatment*	
Definitive local therapy	15 (37)
Local + systemic therapy	25 (61)
Systemic therapy only	1 (2)
Total	41

**Table 2:** Comparison of median survival times.

Variable	Median PFS duration, years	Log-rank test *p* value	Median OS duration, years	Log-rank test *p* value
*Sex*				
Male	5.41	0.21	NA	0.87
Female	3.47		NA	
*Race*				
White	NA	0.38	7.28	0.09
Other	4.18		NA	
*Tumor size*				
≥5 cm	3.33	0.008	NA	0.78
<5 cm	NA		NA	
*Subtype*				
Monophasic	4.17	0.745	7.9	1.00
Biphasic	NA		NA	
Poorly differentiated	5.41		7.22	
*Surgical margin*				
Positive	NA	0.52	NA	0.98
Margin	NA		NA	
*Fusion gene*				
Present	4.02		NA	
*Site*				
Neck	3.47	0.04	7.28	0.27
Other	NA		NA	
*Treatment*				
Definitive local therapy	5.41	0.78	NA	0.80
Local + systemic therapy	4.58		NA	

*Note*. “NA” indicates that median survival was not reached; PFS: progression-free survival; OS: overall survival.

**Table 3:** Univariate Cox proportional-hazards model results.

Variable	PFS	OS
	HR (95% CI)	HR (95% CI)
*Sex*		
Male	0.52 (0.19–1.47)	0.90 (0.25–3.20)
Female		
*Race*		
White	0.65 (0.25–1.71)	4.97 (0.65–37.83)
Other		
*Tumor size*		
≥5 cm	4.69 (1.34–16.38)	1.17 (0.38–3.58)
<5 cm		
*Subtype*		
Biphasic	0.72 (0.24–2.15)	0.097 (0.31–3.07)
Poorly differentiated	0.55 (0.07–4.33)	1 (0.12–8.29)
Monophasic		
*Surgical margin*		
Positive	0.50 (0.60–4.21)	0.97 (0.11–8.69)
Margin		
*Site*		
Neck	2.41 (1.00–5.82)	1.75 (0.64–4.85)
Others		
*Treatment*		
Local + systemic therapy	1.14 (0.45–2.91)	1.16 (0.39–3.46)
Definitive local therapy		

PFS: progression-free survival; OS: overall survival; HR: hazard ratio; CI: confidence interval.

**Table 4:** Summary of overall and progression-free survival rates with other HNSS cohorts.

Study	Cohort size	Major findings
Roth et al.^b^ (1975)	*N* = 22	5-year OS = 22%

Harb et al. (2007)	*N* = 40	5-year OS = 72%
		*Tumor size*
		≥5 cm = 336 months
		<5 cm = 622 months

Crowson et al. (2015)	*N* = 28	Median OS = 56 months
		*Tumor size*
		≥5 cm = 45 months
		<5 cm = 60 months

Wushou et al.^a^ (2015)	*N* = 93	*Treated with surgery*
		5-year OS = 81.4%
		10-year OS = 78.3%
		*Tumor size*
		≥5 cm = undefined
		<5 cm = 30 months

Mallen St. Clair et al. (2016)	*N* = 167	5-year OS = 66%
		*Tumor size*
		≥5 cm = 4.4 years
		<5 cm = undefined

^a^Meta-analysis. ^b^Anterior neck tumors only.
