# Supplementary material for: Synovial Sarcoma of the Head and Neck: A Single Institution Review
Source: Sarcoma. 2017 Jun 5;2017:2016752. doi: 10.1155/2017/2016752 (PMC5474548; doi:10.1155/2017/2016752)
Supplement: Supplementary file 1 — Table S1: Comparison of median PFS and OS times by timing of radiation and chemotherapy in patients with head and neck synovial sarcomas. Table S2: Comparison of median overall survival time by therapeutic regimen in patients with head and neck synovial sarcoma who were diagnosed with recurrent disease. [file 2016752.f1.pdf]

Synovial sarcoma of the head and neck: a single institution review

Vancheswaran Gopalakrishnan, [1] Behrang Amini, [2] Michael J Wagner, [3] Erica N. Nowell, [4] Alexander Lazar, [5] Patrick P. Lin, [6] Robert S. Benjamin, [4] and Dejka M. Araujo[4]

Supplemental materials

Supplemental table S1: Median progression-free and overall survival based on timing of radiation or chemotherapy

	Median PFS (in yrs)	Log-rank p	Median OS (in yrs)	Log-rank p
**Radiation (n=36)**		0.331		0.072
Adjuvant (n=30)	5.41		7.9	
Neodjuvant (n=5)	NA		NA	
Peri-operative (n=1)	2.23		4.26	
**Chemotherapy (n=26)**		0.87		0.118
Neoadjuvant (n=13)	3.95		4.52	
Adjuvant (n=13)	4.58		NA	

Supplemental table S2: Overall survival by choice of therapeutic regimen after recurrence

	N (%)	Median OS[#] (in years)	Log-rank p
Local therapy only	5 (0.25)[a]	3.4	0.643
Local therapy + Additional systemic therapy	9 (0.45)[b]	2.47	
Systemic therapy only	5 (0.25)	2.33	

[a]: 3 patients underwent surgery, 2 underwent surgery plus local radiation therapy
[b]: 8 patients got surgery + adjuvant chemotherapy. 1 patient got surgery + radiation + adjuvant chemotherapy
OS was calculated as the interval between date of diagnosis of recurrent disease to date of death or last contact
